# Wender Utah Rating Scale-25 (WURS-25): psychometric properties and diagnostic accuracy of the Swedish translation

**DOI:** 10.1080/03009734.2018.1515797

**Published:** 2018-10-29

**Authors:** Ioannis Kouros, Niklas Hörberg, Lisa Ekselius, Mia Ramklint

**Affiliations:** Department of Neuroscience, Psychiatry Uppsala University, Uppsala, Sweden

**Keywords:** ADHD, bipolar disorder, borderline personality disorder, Wender Utah Rating Scale

## Abstract

**Objective:** The aim of this study was to examine the psychometric properties and diagnostic accuracy of the Swedish version of the Wender Utah Rating Scale (WURS) in psychiatric patients with similar symptoms but diagnosed with either attention deficit hyperactivity disorder (ADHD), bipolar disorder (BP), and/or borderline personality disorder (BPD).

**Methods:** A total of 121 patients from an outpatient psychiatric clinic for young adults (18–25 years) were diagnosed using the Structured Clinical Interview for DSM Axis I and Axis II (SCID-I and SCID-II), and ADHD was diagnosed using the Schedule for Affective Disorders and Schizophrenia for School-Age Children (K-SADS). WURS were filled in by the participants and compared with a diagnosis of ADHD according to K-SADS.

**Results:** Internal consistency of the WURS was 0.94. The principal component analysis resulted in a three-factor solution that accounted for 61.3% of the variance. The ADHD group had significantly higher mean scores compared to all other groups. The diagnostic accuracy of the WURS was examined using AUC and ROC analysis, and the optimal cut-off score was 39, with a sensitivity of 0.88 and specificity of 0.70, with AUC 0.87, 95% CI 0.80–0.94, PPV 0.59, and NPV 0.92.

**Conclusion:** The psychometric properties of the Swedish WURS were good. For assessment of adult ADHD, in patients with symptoms of emotional instability, impulsivity, and attention problems but of different origins, a somewhat higher cut-off score than the originally suggested was preferable for identification of ADHD.

## Introduction

Attention deficit hyperactivity disorder (ADHD) is mainly characterized by deficiency in sustaining attention, impulsivity, and/or hyperactivity (1). ADHD can be found in about 5% of school-age children (2). Current evidence from follow-up studies of children with ADHD has shown that the disorder persists to a high degree in adulthood, with a prevalence of about 4% (3,4). Diagnosing ADHD in adulthood is challenging due to the need for retrospective recall and due to high rates of other psychiatric disorders with similar symptoms (5–7). Such disorders are bipolar disorder (BP) and borderline personality disorder (BPD), which both present with symptoms that overlap with ADHD symptoms, for example emotional instability and impulsivity. In addition, children diagnosed with ADHD have been reported to have an increased risk of developing BPD in late adolescence (8). Moreover, co-occurrence between ADHD and BP or BPD is reported to be around 20% (9).

In clinical practice, the similarity of the behavioural presentation of ADHD, BP, and BPD constitutes a risk for overlooking the symptoms of one of the other disorders. There is therefore a need for diagnostic instruments with satisfactory discriminant validity that can be used for assessment of ADHD in adults, especially in clinical settings where BP and BPD are also common.

The Wender Utah Rating Scale (WURS) is a self-report instrument that is designed to retrospectively assess childhood ADHD symptoms, based on the Utah criteria (10,11). The scale originally consisted of 61 items. The long form was arbitrarily reduced to the 25 items that showed the greatest mean difference between patients with ADHD and controls. In the original validation of the WURS-25, Ward et al. reported a sensitivity and specificity of 96% for a cut-off score of 36, and a sensitivity of 86% and specificity of 99% for a cut-off score of 46 (11). Both the long and the short form of the English version showed good test–retest reliability and internal consistency (12,13). Both a three-factor and a five-factor model have been suggested for the WURS-25 (14–16). WURS has been translated into several languages, and validation studies have shown similar psychometric properties to those reported by Ward et al. (7,15,17–20). A Swedish translation of WURS is available and frequently used. However, no validation study of the Swedish version has been published.

The aim of this study was to examine the psychometric properties and diagnostic accuracy of the Swedish version of the WURS-25 in patients diagnosed with ADHD, BP, and/or BPD. We hypothesized that the instrument should have similar internal consistency and factor structure to the original English version, that it should be able to discriminate patients with ADHD from patients with BP and/or BPD, and, finally, that it should show adequate diagnostic accuracy for identifying a diagnosis of ADHD in this phenomenologically homogeneous group.

## Material and methods

### Participants

Patients from an outpatient psychiatric clinic for young adults (18–25 years) in Uppsala, Sweden, diagnosed with BPD, ADHD, or BP, were identified from the administrative patient register. Altogether, 759 patients diagnosed with any of the above diagnoses between 1 May 2005 and 31 October 2010 were sent a study invitation to participate in the study by post. Letters were sent to groups of patients on 24 different occasions from 18 August 2008 to 13 May 2011. At the time of investigation, some patients still had ongoing contact with the clinic, whereas others did not. Exclusion criteria were severe psychotic or manic symptoms at the interview appointment. One patient was excluded because of ongoing mania.

**Figure 1.**
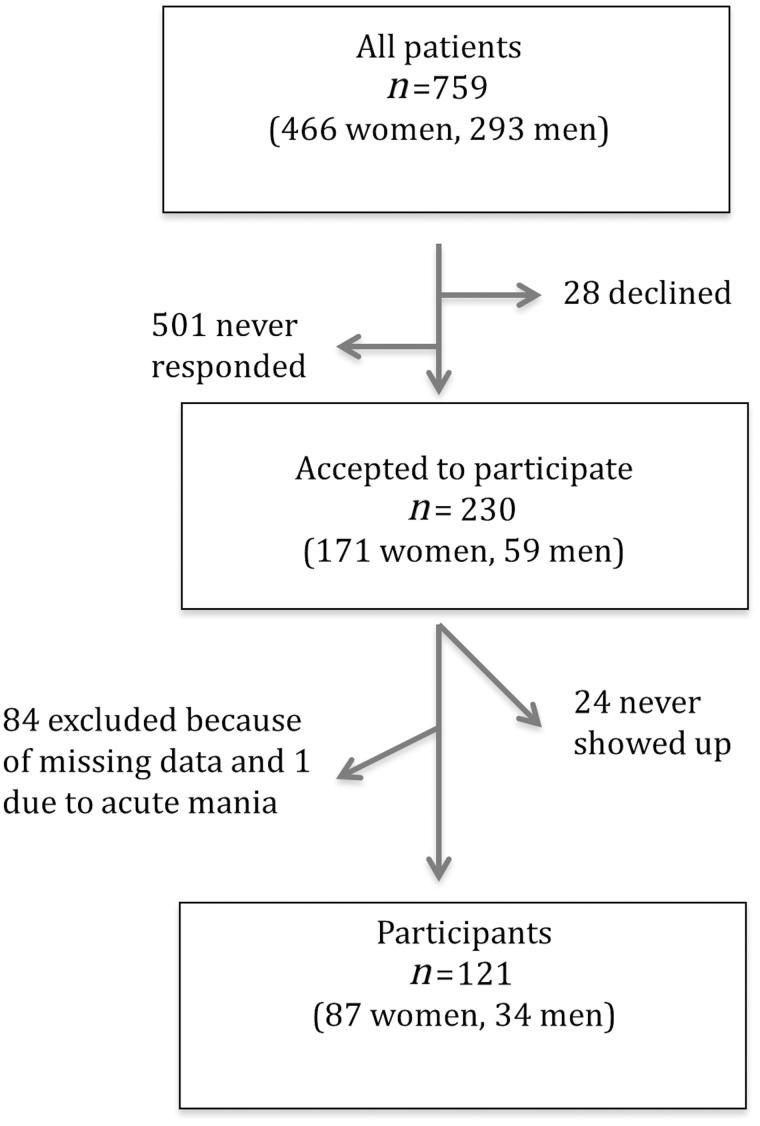
Recruitment to the study.

For a description of the recruitment process, see Figure 1. There were 230 (30%) who agreed to participate, and of these 121 (53%) had no missing data and therefore constitute this study group. After completing diagnostic interviews in those patients whose clinical diagnoses were not based on structured interviews, 20 individuals did not fully meet the criteria for any of the three diagnoses. Therefore this group was called subclinical cases. Thirteen of the subclinical cases had a prior ADHD diagnosis, two had a prior BPD, four had a prior BP, and one had a prior comorbid ADHD and BPD diagnosis, implying that the subclinical group was dominated by subclinical ADHD cases. Within the subclinical cases we estimated the functional impairment according to the Global Assessment of Functioning Scale (GAF) (1). The mean GAF score in the subclinical cases was 62.8 (SD 8.3), compared with 59.8 (SD 11.4) in the clinical cases.

Among the participants, there were 40 (33.1%) who fulfilled criteria for ADHD. Among those 40, there were 19 diagnosed with ADHD alone, eight had a comorbid BPD, eight a comorbid BP, and five fulfilled criteria for all three diagnoses; however, all were brought together into an ADHD group. The other groups were 17 (14.0%) BPD, 30 (24.8%) BP, 14 (11.6%) comorbid BPD and BP, and finally 20 (16.5%) subclinical cases.

### Drop-out analysis

Among all 759 available patients, there were 466 (61.4%) women and 293 (38.6%) men. Among those 230 who participated, there were significantly more women than men (74.3 versus 25.7%; chi-square 23.35; *P* < 0.001). Among our study group, 14.3% were previously diagnosed with BPD, 32.2% with ADHD, and 35.2% with BP.

Comparing the 121 participants with the 109 internal dropouts from the 230 who accepted participation did not show any significant differences in gender or distribution of diagnoses.

### Procedure

The study was cross-sectional, and the participants were interviewed on one or two occasions, depending on the time needed. First, a basic interview about anamnestic, social, and demographic data was performed using a checklist. Subsequently, complementary semi-structured diagnostic interviews were performed in patients whose diagnoses were not based on structured interviews selected for the study; see below. Thereafter, the patients filled in the study questionnaires. Pervasive developmental disorders (PDD) was not a reason for exclusion, but diagnostic criteria were not assessed as part of the study. However, participants filled in the Autism Spectrum Quotient (AQ), and the mean score was 19.3 (median 18, SD 7.0), supporting a low impact of autistic traits in the study group (21).

There were 21 participants who reported on the WURS before the K-SADS interview (Schedule for Affective Disorders and Schizophrenia for School-Age Children) was performed, 44 participants performed both tests on the same day, 53 reported on the WURS after the interview, and the date was missing for three patients. The median time interval between the index test, WURS, and the reference test was zero weeks (SD 51). Patients were grouped in two groups, those with less (*n* = 66) or more (*n* = 55) than 30 days between the two tests, including the three without known date placed in the latter group, and diagnostic accuracy was then examined separately in both groups.

## Assessments

### Structured Clinical Interview for DSM Axis I Clinical Version (SCID-I-CV)

SCID-I-CV is a semi-structured diagnostic interview that provides comprehensive assessment of most psychiatric diagnoses according to DSM-IV (22). The test–retest reliability of SCID-I has previously been shown to be high (kappa =0.84) for bipolar disorders in clinical populations (23). In order to provide a structured way to diagnose BP Not Otherwise Specified (NOS),v, patients reporting elevated or irritable mood with a shorter duration than four days were assessed with all questions about manic symptoms. Patients diagnosed with BP I, BP II, and BP NOS were all included in the BP group.

### Structured Clinical Interview for DSM Axis II (SCID-II)

SCID-II is a semi-structured diagnostic interview for assessment of personality disorders according to DSM-IV (24). The reliability of the SCID-II has been assessed in several studies. Although there is a high variation in the results, recent studies including larger numbers of participants report higher reliability statistics (25,26).

Patients filled in the SCID-II personality questionnaire. General personality disorder criteria and BPD criteria were assessed in all participants by using the SCID-II interview. Patients who admitted symptoms above the cut-off for any other personality disorder were interviewed with all criteria for the disorder. In 13 cases, there were missing data for personality disorders, but not for BPD. During the interview, state effects of axis I symptomatology on the reports of personality functioning was evaluated by follow-up questions. The majority of the participants (*n* = 90) also filled in the Montgomery Åsberg Depression Rating Scale–Self assessment (MADRS-S) with a mean score 19.3 (SD 10.2) (27).

### Schedule for Affective Disorders and Schizophrenia for School-Age Children (K-SADS)

K-SADS is a semi-structured diagnostic interview that examines psychopathology in children and adolescents between the ages of six and 18 years (28). Since there were no other validated interviews in Swedish translation available for assessment of ADHD in adults at the time the study started, the K-SADS was chosen (for these young adults). A list of the DSM-IV criteria that corresponded to the questions in the K-SADS supplement was composed. Only the supplement for ADHD was used, and, based on the information from the interview together with the medical history, all ADHD criteria were assessed. All subtypes were included, ADHD, Attention Deficit Disorder (ADD), and Hyperactivity Disorder without Attention Deficit (HD), all labelled ADHD. The time frame used was ‘childhood’, and the patient was asked to consider if the symptoms had been present before the age of seven years.

### Wender Utah Rating Scale (WURS)

WURS is a Likert-type, self-report scale that is designed to retrospectively assess childhood ADHD symptoms. The WURS has previously shown good internal consistency and a moderate convergent validity when correlated with the 10-item Parents’ Rating Scale (11). Both a three- and a five-factor model have been shown for the WURS-25 (14,16). In the McCann et al. study (14), the three factors were labelled Dysthymia, Oppositional/Defiant Behavior, and School Problems, but only School Problems in childhood distinguished ADHD from non-ADHD patients. Suhr et al. showed a five-factor model consisting of conduct problems, impulsivity problems, mood difficulties, inattention/anxiety symptoms, and poor academic functioning with reasonable internal consistencies (16). In the Suhr et al. study, subscales reflecting impulsivity, inattention/anxiety, and poor academic functioning discriminated ADHD from controls in females (16).

In the original validation of the WURS-25, Ward et al. reported a diagnostic accuracy for the instrument (11). With a cut-off score of 36 both sensitivity and specificity were 96%, whereas for a cut-off score of 46 sensitivity was 86% and specificity 99%. For the Finnish version of WURS-25, a cut-off score of 36 was suggested with a sensitivity of 89% and a specificity of 85% (17).

In this study, participants filled in the 61-item questionnaire, but only the 25 questions known to best discriminate between ADHD and controls were used—the WURS-25.

### Diagnostic reliability

Three medical doctors performed the majority (94%) of all the diagnostic interviews (SCID-I, SCID-II, K-SADS): one specialist in psychiatry (M.R.), one resident in psychiatry (I.K.) and one intern (N.H.). All three had been trained in accordance with the SCID manual by reviewing recorded interviews with a ‘master interviewer’ and by then assessing patients from audio and video recordings, assessing patients in clinical practice, and discussing both performance and appraisal of responses with experienced raters. Four per cent of the interviews were performed by clinicians that previously had shown good inter-rater reliability with the first author.

Inter-rater reliability was calculated for the three main interviewers (M.R., I.K., N.H.) based on six randomly selected SCID-I interviews (13 protocols), mean prevalence-adjusted bias-adjusted kappa (PABAK) 0.95, range 0.91–0.97; nine SCID-II interviews (23 protocols), mean PABAK 0.85, range 0.79–0.88; and four K-SADS interviews (11 protocols), mean PABAK 0.72, range 0.64–0.81. Not all diagnoses within the K-SADS supplement were assessed, only ADHD. Inter-rater reliability of K-SADS therefore meant agreement upon an ADHD diagnosis or not. Diagnostic reliability was assessed before and during the study as part of regular meetings.

### Statistics

For inter-rater reliability, PABAK statistics were used (29). The internal consistency of the WURS-25 was measured by Cronbach’s alpha. A principal component analysis using varimax rotation was performed on the WURS-25. We retained the number of factors using the scree plot and the Kaiser–Guttman rule (eigenvalues greater than 1.0). Discriminant validity was evaluated by comparing mean scores between different diagnostic groups using one-way ANOVA with Tukey’s *post hoc* test. The previously suggested cut-off scores (36 and 46) were used for calculations of sensitivity, specificity, positive predictive value (PPV), and negative predictive value (NPV) for an ADHD diagnosis. Receiver-operating characteristics (ROC) analyses were used for the area under the curve calculation (AUC). We calculated Youden’s J and applied a minimum score of 0.85 for sensitivity and 0.70 for specificity to choose the optimal cut-off (30).

The study was approved by the regional board of the Ethics Committee of Uppsala.

## Results

The WURS-25 had a high level of internal consistency of 0.94, as determined by Cronbach’s alpha.

The principal component analysis resulted in a three-factor solution that accounted for 61.3% of the variance. The Kaiser–Mayer–Olkin coefficient was 0.92. The three factors were labelled ‘impulsivity/behavioural problems’, ‘inattentiveness/school problems’, and ‘self-esteem/negative mood’. The alpha coefficient for the ‘impulsivity/behavioural problems’ factor was 0.94, for ‘inattentiveness/school problems’ 0.83, and for ‘self-esteem/negative mood’ 0.81 (Table 1).

**Table 1.** The principal component factor analysis of the wender Utah rating scale-25 (WURS-25) in young psychiatric patients (*n* = 121).

	Factor 1: Impulsivity/behavioural problems	Factor 2: Inattentiveness/school problems	Factor 3: Self-esteem/negative mood
	7. Hot- or short-tempered	3. Concentration problems	4. Anxious, worrying
	9. Temper outbursts	6. Inattentive, daydreaming	5. Nervous, fidgety
	11. Stubborn, strong-willed	10. Trouble with stick-to-it-iveness	12. Sad or blue, depressed, unhappy
	15. Disobedient with parents, rebellious	25. Tend to be immature	16. Low opinion of myself
	17. Irritable	51. Overall a poor student	26. Feel guilty, regretful
	20. Moody, ups and downs	56. Trouble with mathematics	
	21. Feel angry	59. Did not achieve up to potential	
	24. Acting without thinking, impulsive		
	27. Lose control of myself		
	28. Tend to be or act irrational		
	29. Unpopular with other children		
	40. Trouble seeing things from someone else’s point of view		
	41. Trouble with authorities, trouble with school		
Eigenvalue	11.643	2.013	1.667
% of variance	46.6	8.0	6.7
Cronbach’s alpha	0.939	0.833	0.809

We compared the mean WURS-25 scores for the individuals that met the criteria for ADHD (*n* = 40) with the mean scores of the individuals with BPD (*n* = 17), BP (*n* = 30), BPD and BP (*n* = 14), and the individuals with subclinical diagnoses (*n* = 20). In all groups there were more female than male participants; in the ADHD group the percentage of females was 65%, in the BPD group 94%, in the BP group 73%, in the BPD and BP group 86%, and in the group with subclinical diagnoses 55%. The ADHD group had significantly higher mean scores on WURS compared to all other groups. Mean WURS scores for all groups are presented in Table 2. There was no statistical difference in the mean WURS scores between males and females (*t* [119] = 0.115, *P* = 0.909).

**Table 2.** Results on WURS-25 in different diagnostic groups, for the 121 participating young adult psychiatric patients. Comparisons of mean scores (standard deviation, SD) using ANOVA, reflecting discriminative validity.

	a) ADHD (*n* = 40) mean (SD)	b) BPD (*n* = 17) mean (SD)	c) BP(*n* = 30) mean (SD)	d) BPD and BP (*n* = 14) mean (SD)	e) Subclinical cases (*n* = 20) mean (SD)	*F*	*P*	*Post hoc*
WURS-25 total	61.7 (19.3)	31.1 (15.5)	28.1 (20.9)	33.0 (12.9)	35.3 (16.5)	19.099	<0.001	a > b,c,d,e
Impulsivity/behavioural problems	31.9 (11.7)	15.0 (8.6)	13.9 (12.2)	16.3 (8.3)	15.4 (10.3)	16.289	<0.001	a > b,c,d,e
Inattentiveness/school problems	18.0 (4.9)	9.4 (5.3)	7.2 (6.4)	8.2 (5.0)	11.25 (6.0)	19.789	<0.001	a > b,c,d,e
Self-esteem/negative mood	11.8 (5.0)	6.7 (4.2)	7.0 (5.4)	8.5 (4.4)	8.6 (4.0)	5.772	<0.001	a > b,c
Number of axis I diagnoses	2.3	2.9	1.9	1.9	2.0	1.992	0.100	
Number of axis II diagnoses	1.5	2.5	0.5	1.9	0.8	6.827	<0.001	b > c,d,e

ADHD: attention deficit hyperactivity disorder; BPD: borderline personality disorder; BD: bipolar disorder; WURS-25: Wender Utah Rating Scale-25.

**Figure 2.**
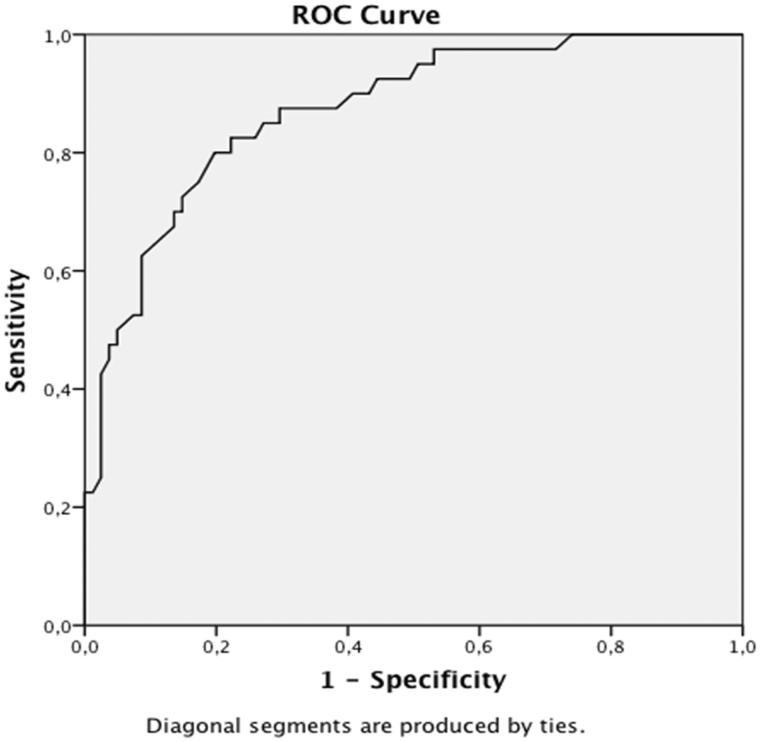
Receiver-operating characteristics (ROC) analysis for the WURS-25 in a clinical psychiatric sample (*n* = 121) diagnosed with ADHD, BD, BPD, or subclinical cases.

The diagnostic accuracy of the WURS-25 was examined using AUC and ROC analysis (Figure 2 and Table 3). The optimal cut-off score was 39, with a sensitivity of 0.88 and specificity of 0.70, with AUC 0.87 and 95% CI 0.80–0.94. We conducted separate analyses for the participants who were interviewed with K-SADS within a month from completing the WURS (*n* = 66), and the group with a time period longer than 30 days (*n* = 55). Sensitivity, specificity, PPV, and NPV for both the original and the optimal cut-off are presented in Table 2.

**Table 3.** Diagnostic accuracy of the WURS-25 in identifying ADHD; results from those who performed the WURS-25 within 30 days or more than 30 days after the K-SADS interview are presented separately.

	All WURS-25 (*n* = 121)				WURS-25 performed within 30 days from the K-SADS (*n* = 66)				WURS-25 performed more than 30 days from the K-SADS (*n* = 55)			
WURS cut-off	36	38	39	46	36	38	39	46	36	38	39	46
Sensitivity	0.90	0.88	0.88	0.80	0.95	0.95	0.95	0.95	0.85	0.80	0.80	0.65
Specificity	0.59	0.68	0.70	0.80	0.63	0.69	0.69	0.76	0.54	0.63	0.66	0.86
PPV	0.52	0.58	0.59	0.66	0.56	0.60	0.60	0.66	0.48	0.52	0.54	0.70
NPV	0.92	0.92	0.92	0.89	0.96	0.97	0.97	0.97	0.88	0.86	0.87	0.83

K-SADS: Schedule for Affective Disorders and Schizophrenia for School-Age Children; NPV: negative predictive value; PPV: positive predictive value; WURS-25: Wender Utah Rating Scale-25.

Since most of the subclinical cases in our study were patients with subclinical ADHD symptoms a complementary analysis considering a minimum of five instead of six criteria for fulfilling an ADHD diagnosis was conducted. Using five criteria as threshold the number of individuals fulfilling the ADHD diagnosis increased from 40 to 53. The sensitivity and specificity for a cut-off score of 36 points was 0.87 and 0.66, respectively, and for a cut-off score of 38, respectively 0.81 and 0.72. The optimal cut-off score was 39 with a sensitivity of 0.81 and specificity of 0.76, with AUC 0.87, and 95% CI 0.81–0.93.

## Discussion

The psychometric properties of the Swedish version of the WURS-25 were satisfactory and mirrored the psychometric properties of the questionnaire in other languages. The diagnostic accuracy was good in young adult psychiatric patients with ADHD, BP, and/or BPD, considering that these diagnoses express similar symptomatology.

The WURS-25 showed excellent internal consistency in accordance with prior studies (11,18). Moreover, the three-factor model was similar to the three-factor models previously presented (14,15). The ‘inattentiveness/school problems’ scale is very similar to the ‘school/work problems’ factor presented in the McCann factor analysis. The other two factors in our analysis are quite similar to the factors presented by Caci et al. as ‘impulsivity/temper’ and ‘mood/self-esteem’ (15). One difference though is that items 40 (‘Trouble seeing things from someone else’s point of view’) and 41 (‘Trouble with authorities, trouble with school’) load on the ‘impulsivity/behavioural problems’ factor in our study. This difference might be attributed to the difference in the study populations as we studied clinical cases with a high psychiatric burden, whereas other studies included university students, relatives of children referred to an ADHD clinic, or adults referred to an ADHD clinic but not yet diagnosed (14,15,18).

The items of hyperactivity and inattention loaded on two different factors, which was also the case in previous studies (15,18). The ADHD group had significantly higher scores on all three subscales. The ‘self-esteem/negative mood’ subscale did not significantly differentiate the ADHD group from the ‘borderline/bipolar comorbidity’ and ‘subclinical diagnosis’ groups (Table 2). Most of the individuals in the subclinical diagnosis group had an ADHD diagnosis prior to entering the study, and this could explain the high mean scores of that group. In the study by Kivisaari et al., the ‘mood difficulties’ domain differentiated poorly between the ADHD group and the dyslexia group (17). Symptoms of negative mood and low self-esteem are not specific to the DSM-IV criteria for ADHD and were common in all the study groups.

Since WURS-25 is a screening tool it is important that its sensitivity is high. The optimal score of 39 in the present study is lower than the cut-off that was suggested in the original validation study (11). However, it is higher than the score of 36 that is currently arbitrarily suggested as cut-off for the Swedish version of WURS-25. Kivisaari et al. suggested a cut-off score of 37 when comparing an ADHD group with a pooled group of controls and patients with dyslexia (17). The results of this study showed, however, that when performing assessment of adult ADHD in patients with different diagnoses but similar symptoms, the cut-off score of 39 was preferable to the cut-off score of 36. This is also supported by previous studies where patients with BPD or BP score higher on WURS; for example, in a study of the Korean version of WURS patients with BP scored significantly higher than controls, and, in a study using the Italian version of the WURS-25, significantly more patients with BPD scored higher than 46 compared to patients with other personality disorders and controls (5,31). When five instead of six criteria for ADHD were used as threshold the results were similar, while sensitivity decreased and specificity increased, but only slightly. Using the minimum of six criteria might have excluded some less severe cases of ADHD that could have changed the diagnostic accuracy in a substantial way. It does not seem, however, that this was the case. We also analysed the results of those that completed the WURS within a month of the K-SADS interview and compared them to the results of those that did not complete the WURS within the same period of time. The results showed slightly lower sensitivity and specificity if the time between the tests increased. There are several potential reasons for this change over time. There could be recall bias, since if you struggle with both ADHD and other axis I disorders, such as depression, it might be hard to address symptoms and impairment related to ADHD alone. Being asked later, when axis I comorbidity has improved, might explain the difference over time.

This study has several limitations. One is the lack of validated interviews in Swedish for assessing adult ADHD. We therefore had to use the K-SADS, which is an interview for children and adolescents. Furthermore, we did not use the screening questions, only the questions from the K-SADS supplement to evaluate ADHD. This might have affected the estimation of the true ADHD cases. However, all ADHD criteria were explicitly evaluated based on both the interview and medical history. Both WURS and K-SADS are based on the same retrospective data, the patient’s recall from childhood. In many cases a parent had reported childhood symptoms, but this was not mandatory, and for most cases we relied on the patient as the only informant. Another limitation is the use of clinical data, collected during the diagnostic assessments in the clinic. However, the number of diagnostic interviews performed by other clinicians was very limited, and in half of them the diagnosticians were known to be reliable. Inter-rater reliability was calculated on a limited number of interviews, and for K-SADS the agreement was good, but not excellent. A further limitation is that according to the DSM-IV criteria for ADHD the presence of some inattentive and hyperactive/impulsive symptoms before the age of seven is needed, but it is not a requirement for all symptoms to have been present before seven years of age. In accordance with the K-SADS interview structure we asked about the onset at the same time as we evaluated each criterion. This might have caused an underestimation of the true ADHD cases. However, our impressions from the interviews were that most symptoms were present early in life.

Strengths include the presentation of separate analyses for those questionnaires filled in more or less than 30 days apart from the reference standard. The absence of a healthy control group might have revealed even greater differences between groups and supported discriminant validity even more. However, our aim was to investigate the psychometric properties of the WURS-25 in a clinical setting considering the difficulties in the assessment of adult ADHD in patients with similar symptoms.
